# An Unusual Cause of Persistent Tachycardia: Atypical Neuroleptic Malignant Syndrome

**DOI:** 10.7759/cureus.36428

**Published:** 2023-03-20

**Authors:** Sanchit Duhan, Bijeta Keisham, Anubhav Jain

**Affiliations:** 1 Internal Medicine, Sinai Hospital of Baltimore, Baltimore, USA; 2 Department of Cardiology, Ascension Genesys Hospital, Grand Blanc, USA

**Keywords:** drug-induced acute kidney injury, mood stabilizer, atypical neuroleptic malignant syndrome, lithium intoxication, sinus tachycardia

## Abstract

Neuroleptic malignant syndrome (NMS) is a rare, life-threatening emergency caused more commonly by typical antipsychotics. However, unusual presentations of NMS are intermittently reported with the use of atypical antipsychotics. We present the case of a 42-year-old gentleman with schizoaffective and bipolar disorder who was admitted for change in mentation and lithium toxicity. His mentation did not improve despite being dialyzed and the resolution of lithium level to baseline. He developed persistent tachycardia and hyperthermia, initially attributed to *Streptococcal* infection. But despite appropriate antibiotic therapy, his clinical symptoms did not improve. An extensive workup for his neurological symptoms, including lumbar puncture, 5-hydroxy indole acetic acid urine test, and brain magnetic resonance imaging, was inconclusive of any underlying etiology. Given the suspicion of atypical NMS, bromocriptine 2.5 mg three times daily was initiated. This led to the gradual resolution of his symptoms and a return to his baseline mental status. Diagnosing atypical NMS can be challenging and must be differentiated from similar disorders. Lithium toxicity can predispose patients to develop NMS.

## Introduction

Neuroleptic malignant syndrome (NMS) is a life-threatening condition with a reported incidence of 0.02% to 3% in patients on antipsychotic medications [1]. NMS is characterized by high fever, tachycardia, muscular rigidity, altered mental status, and laboratory findings, including creatinine phosphokinase (CPK) elevation and often elevated white blood cells (WBCs). It is more commonly reported with high-potency antipsychotics like haloperidol [1]. Atypical antipsychotics have lower extrapyramidal symptoms (EPS) rates and are less commonly associated with NMS [1]. Other causative agents include antiemetic medications such as promethazine and metoclopramide [1]. First-generation antipsychotics have a high affinity to dopamine D2 receptors leading to strong antagonistic activity and a higher risk of EPS. Atypical antipsychotics have a lower affinity to D2 receptors and a higher affinity for serotonin and norepinephrine receptors, decreasing their risk of NMS [2]. However, rare cases of aripiprazole-induced NMS have been intermittently reported [1,3-5].

Many cases caused by atypical antipsychotics differ from the classically described NMS [6]. Hence, it was hypothesized that there are atypical forms of NMS depending on the different pharmacologic properties of atypical antipsychotics. It is established that no antipsychotic is free from the risk of NMS [6]. The diagnosis of atypical NMS is challenging. To aid in identifying the spectrum of NMS and the underlying risk factors, we present this case of atypical NMS secondary to aripiprazole use precipitated by lithium toxicity.

## Case presentation

A 42-year-old African American man presented to the hospital with lethargy and a change in mental status for three days. His medical history was significant for borderline personality disorder, opioid use disorder, and schizoaffective disorder. The patient had taken more doses of lithium than usual to control his auditory hallucinations. He could not provide information regarding the number and period of medication overdosing. His home medications included aripiprazole 15 mg once daily, benztropine 1 mg twice daily, hydroxyzine hydrochloride 50 mg once daily, buprenorphine 8 mg daily, and lithium 300 mg twice daily.

**Figure 1:**
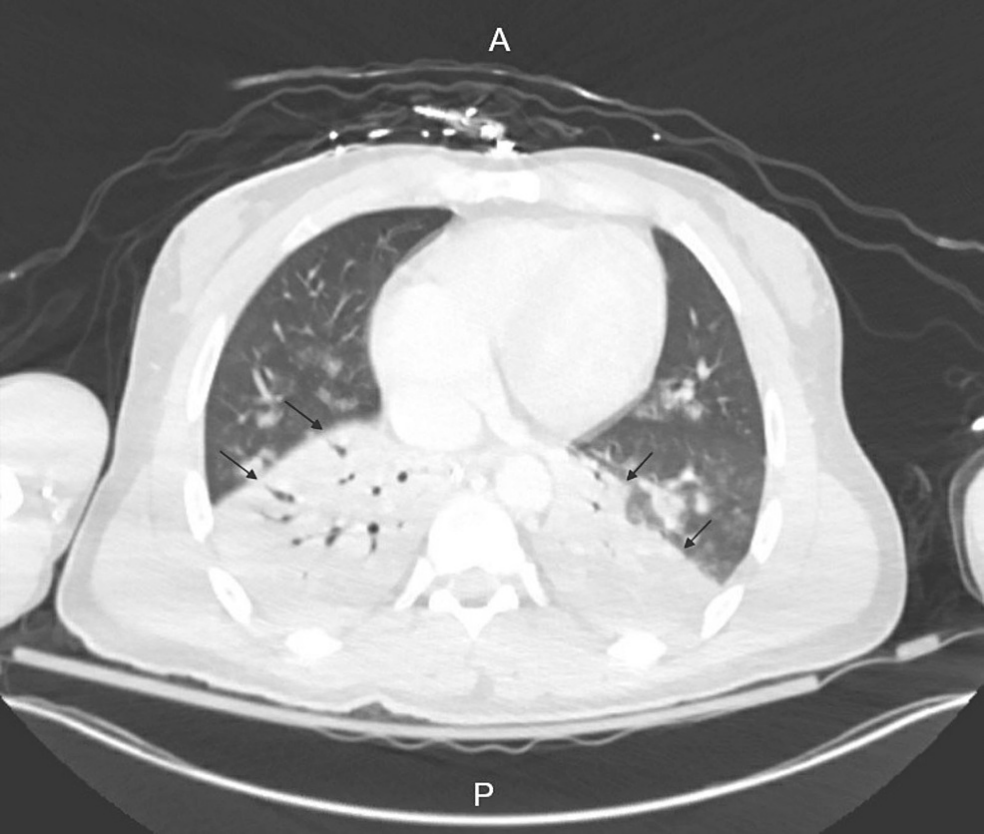
Chest computed tomography scan showing multilobar pneumonia, with greatest consolidation in lower lung lobes (black arrows) A, anterior; P, posterior

On presentation, his vitals were within normal limits except for mild tachycardia. He was drowsy but easily arousable and alert-oriented to time, place, and person. His laboratory panel was remarkable for elevated blood urea nitrogen (BUN), creatinine, and lithium level (Table 1). Complete blood count, urine toxicology, urinalysis, and respiratory viral panel were within normal limits. Electrocardiogram (EKG) showed normal sinus rhythm, and troponin level was normal. N-terminal prohormone brain natriuretic peptide (NT-proBNP) was slightly raised at 503 pg/mL (normal range: <125 pg/mL) secondary to acute kidney injury (AKI). The patient was treated with intravenous (IV) normal saline at 200 cc/hr for lithium toxicity. On day 2, he started feeling short of breath. His mentation significantly worsened to a complete loss of consciousness. He could only mildly withdraw from pain and had intact cranial reflexes, including corneal, pupillary, cough, and gag reflexes. His oxygen saturation dropped to 92% (normal range: 95%-100%), and he developed a fever and hypotension. His tachycardia worsened, and his WBC count decreased to below normal (leukopenia), indicating a septic shock. The AKI resolved, but the lithium level was still elevated. A computed tomography (CT) scan showed multilobar pneumonia (Figure 1). CT head did not show any acute abnormality. He required a high-flow nasal cannula for oxygenation and was empirically treated with ceftriaxone and azithromycin. The nephrology department was consulted due to a suboptimal decrease in lithium levels. Given the patient's change in mentation, there was a concern about the syndrome of irreversible lithium-effectuated neurotoxicity (SILENT). The IV fluids were increased to 250 cc/hr. On day 3, he remained febrile and tachycardic. He again developed an AKI with a rise in creatinine. His lithium levels were still elevated. The patient's urine *Streptococcal pneumoniae* antigen test was positive. Other chemistries included a normal thyroid-stimulating hormone (TSH) and parathyroid hormone (PTH). A plan for dialysis was initiated to treat lithium toxicity in the setting of non-resolution with fluids. The antibiotics were narrowed to ceftriaxone 1 g daily to treat *Streptococcal* infection. Post dialysis, the lithium levels normalized. However, the patient's mentation remained the same. Following a behavioral health consultation, the patient's aripiprazole dose was decreased to 5 mg daily, and hydroxyzine was held to reduce the sedative effects of these medications. His benztropine and buprenorphine were continued at home as there were no identifiable contra-indications. On day 4, the patient's oxygen requirement improved to only requiring a nasal cannula. However, he continued to be tachycardic and febrile. His AKI worsened, but lithium levels and WBC counts improved to normal limits.

**Table 1:** Patient's laboratory values and vital signs during hospitalization WBC, white blood cell; CPK, creatinine phosphokinase; BUN, blood urea nitrogen; HR_max_,_ _maximum heart rate; T_max_, maximum temperature

Days since admission	Lithium level (normal range: 0.6-1.2 mEq/L)	WBC count (normal range: 4,500-11,000 cells/mm^3^)	CPK (normal range: 39-308 IU/L)	BUN (normal range: 7-20 mg/dL)	Creatinine (normal range: 0.5-1.3 mg/dL)	HR_max _(normal range: 60-100 beats/min)	T_max_ (°C) (fever >38.0 °C)
1	3.00	9.68	-	31	1.61	102	37.2
2	2.30	2.33	-	20	0.79	114	38.2
3	1.80	3.76	-	10	1.5	122	39.1
4	1.10	6.30	-	23	1.67	116	38.7
5	-	11.03	-	22	1.26	105	38.2
6	0.50	13.23	-	14	1.08	108	38.5
7	-	10.09	319	18	0.81	104	38.8
8	-	9.93	129	20	0.72	111	39.0
9	-	9.23	119	21	1.04	99	37.4
10	-	9.03	207	20	0.81	86	37.2

**Figure 2:**
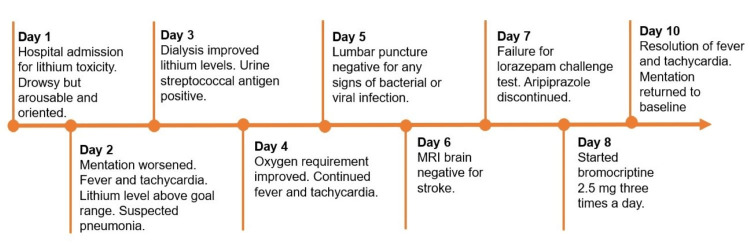
Timeline of significant events during hospitalization MRI, magnetic resonance imaging

On day 5, his mentation remained the same. His physical exam was remarkable for mild bilateral upper extremity and neck rigidity, which spontaneously resolved. He remained tachycardic and febrile. His oxygen requirement further improved, but his WBC counts again mildly increased. Human immunodeficiency virus (HIV) antigen/antibody and syphilis venereal disease research laboratory (VDRL)/enzyme immunoassay (EIA) tests were negative. An electroencephalogram (EEG) was unremarkable for any epileptiform activity. Given the suspicion of meningitis, a lumbar puncture was done. However, cerebrospinal fluid (CSF) studies showed negative results for bacterial, viral, fungal, and parasitic infections. On day 6, the brain's magnetic resonance imaging (MRI) was done to rule out any stroke that showed no abnormality. On day 7, due to an unclear reason for prolonged altered mentation despite extensive workup, a suspicion of catatonia arose due to underlying psychiatric history. The patient was given a lorazepam challenge with two doses of IV lorazepam, 2 mg and 1 mg, respectively, 30 minutes apart. However, he did not show any improvement. Other differentials included atypical NMS and serotonin syndrome. Aripiprazole was discontinued. A CPK level was obtained, which was mildly elevated. A 24-hour urine 5-hydroxy indole acetic acid test was requested to help estimate the serotonin levels in the body, which was normal. On day 8, upon consultation with the neurocritical care department, the patient was started on bromocriptine 2.5 mg every eight hours for possible atypical NMS. On day 9, the patient's mentation slightly improved during the early morning hours. However, by the end of the day, he was again unconscious. His tachycardia improved, and he did not have any febrile episodes. Bromocriptine was continued. On day 10, the patient's mentation improved to being alert and awake with the resolution of tachycardia and fever. Diagnosis of atypical NMS was established. On day 11, aripiprazole was restarted at 5 mg daily to assess his tolerability. The patient received seven days of bromocriptine 2.5 mg three times daily. During the rest of the hospital stay, he was intermittently agitated, which was deemed secondary to residual encephalopathy. However, at the time of his discharge on day 18, his mentation improved to baseline (Figure 2). He was discharged with low-dose aripiprazole 5 mg twice daily. His lithium was discontinued, and further discussion about initiation or alternative medication was planned in a close follow-up outpatient with a psychiatrist.

## Discussion

Lithium is the first-choice mood stabilizer in bipolar disorders. Co-administration of lithium with antipsychotic medications can increase the risk of NMS [7]. Dehydration and lithium-induced renal failure can also exacerbate the condition [7]. Lithium alone can be responsible for causing NMS at toxic levels [7]. We believe a component of all these factors played a role in our patient. In such cases, carbamazepine can be used as a mood stabilizer to prevent the relapse of bipolar disorder [7]. However, given the persistently altered mentation in our case, we held off using carbamazepine to minimize drug interactions.

An inflammatory state such as pneumonia in our patient is another risk factor for the onset of NMS [7]. However, after the symptoms of NMS disappeared in our patient post bromocriptine, his agitation increased, and restarting a lower dose of aripiprazole without concurrent lithium helped improve his symptoms. The atypical NMS in our patient was presumed due to a combination of lithium toxicity and atypical antipsychotic use. The patient was on aripiprazole for a prolonged period (more than five years) and had reasonable control of his symptoms with this medication. Since the underlying lithium toxicity was corrected, we decided to re-introduce the same home medication at a reduced dose while he was still in the hospital under close observation. A gradual initiation of lithium was planned in an outpatient setting after assessing his tolerability to low-dose aripiprazole.

The criteria for diagnosis of NMS are not uniform. Multiple criteria have been proposed, each with a different critical value required to confirm the diagnosis [8]. However, there is still no consensus regarding using clinical features for establishing a diagnosis, and most criteria do not include the empiric diagnosis used in clinical practice [9]. The diagnostic and statistical manual of mental disorders (DSM)-V criteria are commonly used. According to DSM-V, a diagnosis of NMS requires all three significant criteria (exposure to a dopamine-blocking agent, severe muscular rigidity, and fever) and at least two of the other criteria (diaphoresis, dysphagia, tremor, incontinence, altered level of consciousness, mutism, tachycardia, elevated or labile blood pressure, leukocytosis, and elevated CPK) [10]. But given the prevalence of cases where these criteria are unmet, there is a need for revision. Due to a lack of severe rigidity, our patient did not meet the DSM-V criteria. He only had mild rigidity noted by a single physician on an isolated occasion. This could have been an observer bias. Hence, we characterized our case as an atypical NMS. The lack of common symptoms, including significantly elevated CPK and high fevers, made diagnosing NMS difficult in our case.

An explanation of these atypical cases could be a result of the vigilance of physicians who diagnose the patients in the early stages of NMS before the symptoms are established. Another possible explanation is that the use of atypical antipsychotics has a different NMS presentation. In any circumstance, an early diagnosis and treatment can be lifesaving for the patient [11]. A comprehensive metabolic panel including electrolytes, serum creatinine, CPK, urinalysis for myoglobinuria, and blood gas to look for metabolic acidosis can aid in the diagnosis. Leukocytosis can range from 10,000 to 40,000 cells/mm^3^ [12].

In some studies, the mean age of NMS patients is around 40 years [11]. It is more common in men as they are likely to receive higher doses of neuroleptics secondary to more prevalence of positive symptoms [11]. The literature suggests the possibility of genetic risk factors and emphasizes obtaining the patients' family history before starting neuroleptic medications [11]. The first-time use of neuroleptics is a risk factor for precipitating NMS [11]. Evidence suggests that using a single neuroleptic agent instead of multiple agents is associated with decreased risk of NMS [11]. In most cases, NMS appears within one week of neuroleptic initiation. However, rare instances with a 26- to 38-year latency period have also been noted [11].

The management of NMS depends on the severity of the illness. Severe cases need to be admitted to the intensive care unit. Some possible complications of untreated NMS include renal failure, coma, or rhabdomyolysis. The most crucial step in treatment is the discontinuation of the offending agent. The patient is treated with supportive therapy, including cooling and correcting electrolytes and volume deficits. More severe cardiac arrhythmias and respiratory failure cases require antiarrhythmics and mechanical ventilation. Some evidence also suggests that empiric treatment of severe cases with pharmacotherapy may reduce associated morbidity and mortality. Bromocriptine is a dopamine agonist that can reverse the hypodopaminergic state. Benzodiazepines can be used to treat agitation. Another agent is dantrolene which can be used intravenously, and oral forms can be used in less severe cases. In refractory patients, electroconvulsive therapy has been reported to be effective [12].

Some disorders similar to NMS include serotonin syndrome and catatonia. Serotonin syndrome also presents with altered mental status, hyperthermia, autonomic dysfunction, and neurological manifestations. However, unlike NMS, serotonin syndrome is characterized by hyperreflexia and myoclonus. Also, a history of serotonergic medication use will be present. Despite the history of serotonergic medication use in our case, the classic symptoms of serotonin syndrome were missing, which helped rule out the diagnosis. Catatonia presents with immobility, rigidity, mutism, staring, grimacing, waxy flexibility, echolalia, echopraxia, and refusal to eat [10]. These symptoms could help differentiate these diagnoses from typical NMS. However, as seen in our case, the atypical NMS can have various symptoms, making it difficult to diagnose. An extensive workup to rule out the possible etiologies might be required.

## Conclusions

The case highlights the adverse drug reactions during the simultaneous use of lithium with antipsychotics, especially in the setting of renal failure. Diagnosing NMS can be challenging, and high suspicion in a patient with underlying risk factors and altered mental status can be lifesaving. Atypical antipsychotics should be used as cautiously as typical antipsychotics. A close outpatient follow-up to assess the tolerability of medications and gradual initiation of therapies could help prevent adverse events.
